# Integrated perinatal mental health care: a national model of perinatal primary care in vulnerable populations

**DOI:** 10.1017/S1463423618000348

**Published:** 2018-06-18

**Authors:** Kimberly C. Lomonaco-Haycraft, Jennifer Hyer, Britney Tibbits, Jennifer Grote, Kelly Stainback-Tracy, Claire Ulrickson, Alison Lieberman, Lies van Bekkum, M. Camille Hoffman

**Affiliations:** 1 Department of Integrated Behavioral Health, Department of Psychiatry and General Internal Medicine, Denver Health & Hospital Authority, University of Colorado School of Medicine, CO, USA; 2 Department of Obstetrics & Gynecology and Psychiatry, Denver Health & Hospital Authority, University of Colorado School of Medicine, CO, USA; 3 Denver Department of Public Health, CO, USA; 4 Department of Psychiatry, Denver Health & Hospital Authority, University of Colorado School of Medicine, CO, USA

**Keywords:** integrated care, perinatal mental health

## Abstract

**Introduction:**

Perinatal mood and anxiety disorders (PMADs) are the most common complication of pregnancy and have been found to have long-term implications for both mother and child. In vulnerable patient populations such as those served at Denver Health, a federally qualified health center the prevalence of PMADs is nearly double the nationally reported rate of 15–20%. Nearly 17% of women will be diagnosed with major depression at some point in their lives and those numbers are twice as high in women who live in poverty. Women also appear to be at higher risk for depression in the child-bearing years. In order to better address these issues, an Integrated Perinatal Mental Health program was created to screen, assess, and treat PMADs in alignment with national recommendations to improve maternal–child health and wellness. This program was built upon a national model of Integrated Behavioral Health already in place at Denver Health.

**Methods:**

A multidisciplinary team of physicians, behavioral health providers, public health, and administrators was assembled at Denver Health, an integrated hospital and community health care system that serves as the safety net hospital to the city and county of Denver, CO. This team was brought together to create a universal screen-to-treat process for PMAD’s in perinatal clinics and to adapt the existing Integrated Behavioral Health (IBH) model into a program better suited to the health system’s obstetric population. Universal prenatal and postnatal depression screening was implemented at the obstetric intake visit, a third trimester prenatal care visit, and at the postpartum visit across the clinical system. At the same time, IBH services were implemented across our health system’s perinatal care system in a stepwise fashion. This included our women’s care clinics as well as the family medicine and pediatric clinics. These efforts occurred in tandem to support all patients and staff enabling a specially trained behavioral health provider (psychologists and L.C.S.W.’s) to respond immediately to any positive screen during or after pregnancy.

**Results:**

In August 2014 behavioral health providers were integrated into the women’s care clinics. In January 2015 universal screening for PMADs was implemented throughout the perinatal care system. Screening has improved from 0% of women screened at the obstetric care intake visit in August 2014 to >75% of women screened in August 2016. IBH coverage by a licensed psychologist or licensed clinical social worker exists in 100% of perinatal clinics as of January 2016. As well, in order to gain sustainability, the ability to bill same day visits as well as to bill, and be reimbursed for screening and assessment visits, continues to improve and provide for a model that is self-sustaining for the future.

**Conclusion:**

Implementation of a universal screening process for PMADs alongside the development of an IBH model in perinatal care has led to the creation of a program that is feasible and has the capacity to serve as a national model for improving perinatal mental health in vulnerable populations.

## Introduction

Numerous national organizations (American College of Obstetricians & Gynecologists, United States Preventive Services Task Force, American Academy of Pediatrics) have endorsed mental health screening during the perinatal period in an effort to improve pregnancy outcomes, such as preterm birth and low birth weight, as well as to improve long-term maternal–child health and wellness (Colorado Department of Public Health and Environment, 2015; Committee on Obstetric Practice, 2015; Siu *et al*. 2016). Indeed, during the perinatal and first-year postpartum period, suicide, and self-harm are the largest contributors to maternal mortality in the state of Colorado (Gaynes *et al.* 2005; Grote, Zuckoff, Swartz, Bledsoe & Geibel, 2007; Metz *et al*. 2016; Wisner *et al.* 2013). Nationally, in 2009 and 2011, the maternal mortality rate is ~17.8 deaths per 100 000 live births (Centers for Disease Control and Prevention, 2017a; 2017b). The lack of screening for perinatal mood and anxiety disorders (PMADs) is a missed opportunity to serve a vulnerable and more connected population. Imagine if our systems had failed to do routine screenings for gestational diabetes? Integrated women’s care, building off the model of Integrated Primary Care allows for a more robust service that works to engage women in the moment with a holistic mind/body approach to pregnancy and the postpartum period. In addition, women of child-bearing age often interact with the health care system only during pregnancy and the postpartum period and many use perinatal care as primary care (Mbuagbaw *et al*. 2015; Agency for Healthcare Research and Quality, 2016).

Obstetrics and gynecology patients have been found to be nearly four times more likely to follow up with behavioral health treatment when services are offered at the same clinic compared with being referred elsewhere (Byatt *et al*. 2013; Melville *et al*. 2014; Poleshuck and Woods, 2014). In a randomized trial of ‘collaborative depression care’ based in obstetrics and gynecology clinics, the largest impact was found for women who were socially disadvantaged (Katon *et al*. 2015). Standard perinatal care involves scheduled visits that increase in frequency as pregnancy advances; an inpatient admission for labor and delivery and a brief inpatient postpartum stay; and one to two postpartum visits scheduled in the first six to eight weeks after birth. The numerous resources are devoted to perinatal care that address maternal–fetal physical health, thus providing multiple opportunities to engage women in care for their mental health needs (Poleshuck and Woods, 2014). Internationally, the screening rates are routinely higher which provides for connection to services within the larger, and often contiguous, system. Due to the lack of a national health care system in the United States, as well as variable insurance coverage, the United States lags behind in screening and caring for vulnerable women during pregnancy. Across the world including countries in Europe, Australia, and Israel, both peri- and postpartum treatment are built in to standards of care (Agency for Healthcare Research and Quality, 2016; National Institute for Health and Care Excellence, 2017). In many cases, women are entitled to care at no cost to them. Many of these standards are due to the existence of national health organizations and single payer insurance coverage, something that does not currently exist in the United States. The screening and treatment protocols vary by country but can include perinatal counseling, physical therapy before and after delivery to improve physical healing, in home support provided by the state, and connection to national health service programs (Beyondblue, 2011; Massachusetts Child Psychiatry Access Program, 2014; Glasser, 2016; Sims, 2016; National Institute for Health and Care Excellence, 2017).

Barriers to specialty mental health treatment exist, especially in vulnerable populations, such as those served at our Federally Qualified Health Center (FQHC). Studies suggest high rates of attrition when patients are referred to mental health care outside of their trusted system (Blount, 2013). Psychosocial obstacles prevent access to care such as poverty, low education, histories of trauma, poor transportation, and occupational issues. In addition, a stigma exists among patients regarding the need for mental health care during pregnancy and the postpartum period (Gunn and Blount, 2009; Blount, 2013; Melville *et al*. 2014). In combination, these factors lead to a large proportion of patients who do not follow through with outside mental health referrals (Kwee and McBride, 2015). Integration of behavioral health into perinatal clinics is a necessary and urgent programmatic improvement to address PMADs, a leading cause of maternal morbidity and mortality. PMADs affect 15–20% of the general population (Howard *et al*. 2014; Centers for Disease Control and Prevention, 2017a; 2017b), and up to double that proportion in vulnerable patient populations (Katon *et al*. 2015), making them the most common perinatal complication (Howard *et al*. 2014). Perinatal populations at FQHCs are ideal candidates for an integrated behavioral and physical care model because of the existing limitation with regard to specialty mental health clinics for an underserved, underinsured population (Committee on Obstetric Practice, 2015).

The complicated health care system that includes federal, state, and county care initiatives can make can make finding and engaging in behavioral health care challenging. For our already vulnerable and under-resourced population, navigating that system is near impossible. While some states have been able to implement statewide protocols (NY, MA), they are in the minority. Women of color and women who live in poverty are less likely to seek out care for depression proactively, as are mothers who have other demands on their time (National Committee for Quality Assurance, 2017). By integrating behavioral health care into the perinatal clinic, seeing patients on the same day in the same space, care is more convenient, less disjointed, in an environment where interaction with behavioral health is normalized, safe, and validated as a standard of care (Melville *et al*. 2014). Based on this information and the critical need for mental health care in our patient population, the objective of this perinatal mental health program is to create an integrated, sustainable infrastructure for the universal screening, assessment, and treatment of PMADs in alignment with national recommendations.

## Description of the population and program

Denver Health Medical Center is a safety net academic medical center in the City and County of Denver, Colorado, with faculty, residents and students from 39 health care disciplines. Denver residents, 25%, or ~150 000 individuals, receive their health care at Denver Health annually. One in three children in Denver, more than 3300 annually, are born at and cared for at Denver Health Hospital and Community Health Care Clinics.

The Integrated Behavioral Health (IBH) program began 10 years ago as part of a psychology internship training program. Currently, the program is a self-sustained department of 13 psychologists and 16 licensed clinical social workers, four certified addictions counselors, and two psychiatrists who provide services to seven primary care community health service clinics, three Ob-Gyn led women’s care clinics, and several medical and surgical specialty clinics throughout the system. Initially funded through grants, the IBH Division for Ambulatory Care Services has been able to demonstrate financial sustainability to the institution through billing changes and advancements in the Medicaid laws that allow for same-day billing for both medical and mental health care.

## The integrated perinatal mental health program

The Denver Health integrated perinatal mental health program was designed to serve all women who receive prenatal care in our hospital system. This multidisciplinary team includes obstetrician gynecologists, maternal fetal medicine sub-specialists, certified nurse midwives, family practitioners, pediatricians, neonatologists, women’s health nurse practitioners, physician assistants, psychiatrists, psychologists, licensed clinical social workers, nurses, medical assistants, and front desk clerks. Other community agencies involved in the development of our screen-to-treat process included: Denver Public Health and Colorado Department of Public Health and the Environment, and the health system’s LEAN department.

The perinatal clinics serve a diverse population that is a predominantly minority population with a majority of women identifying as Hispanic, living below the national poverty line, with lower health literacy and who are either uninsured or insured by Medicaid. The rationale, upon which the program was built are as follows: (1) prenatal care involves an average of eight encounters during pregnancy plus one to two encounters in the first six to eight weeks postpartum. Therefore, women are interacting with the health care system at regularly scheduled intervals; (2) numerous opportunities exist to address mental health during these multiple scheduled appointments over the course of the perinatal period especially in vulnerable populations; (3) Screening, assessment, treatment, and follow-up of PMADs may lead to improved overall maternal–child health by way of improving pregnancy outcomes (decreasing preterm birth and low birth weight), women’s mental health over the life-span, reducing familial toxic stress, and improving/impacting infant and child mental and physical health and neurodevelopment (Muzik and Brorvska, 2010; Kendig *et al*. 2017); (4) universal screening of all pregnant and postpartum women is now recommended by multiple societies including: The American College of Obstetricians & Gynecologists, The Council on Patient Safety in Women’s Health Care, The United States Preventive Services Task Force, The Agency for Research Health & Quality, The American Academy of Pediatrics, The American Psychiatric Association, and the Health Resources and Services Administration. Screening all patients for depression is, as of 2017, a HEDUS quality measure requirement (National Committee for Quality Assurance, 2017); (5) women, especially from low income communities and communities of color are more likely to receive mental health care from their primary care or community health setting, thus providing an opportune time to engage them in treatment that might otherwise be culturally stigmatized.

## Universal screening and assessment and treatment of a positive screen

**Fig. 1:**
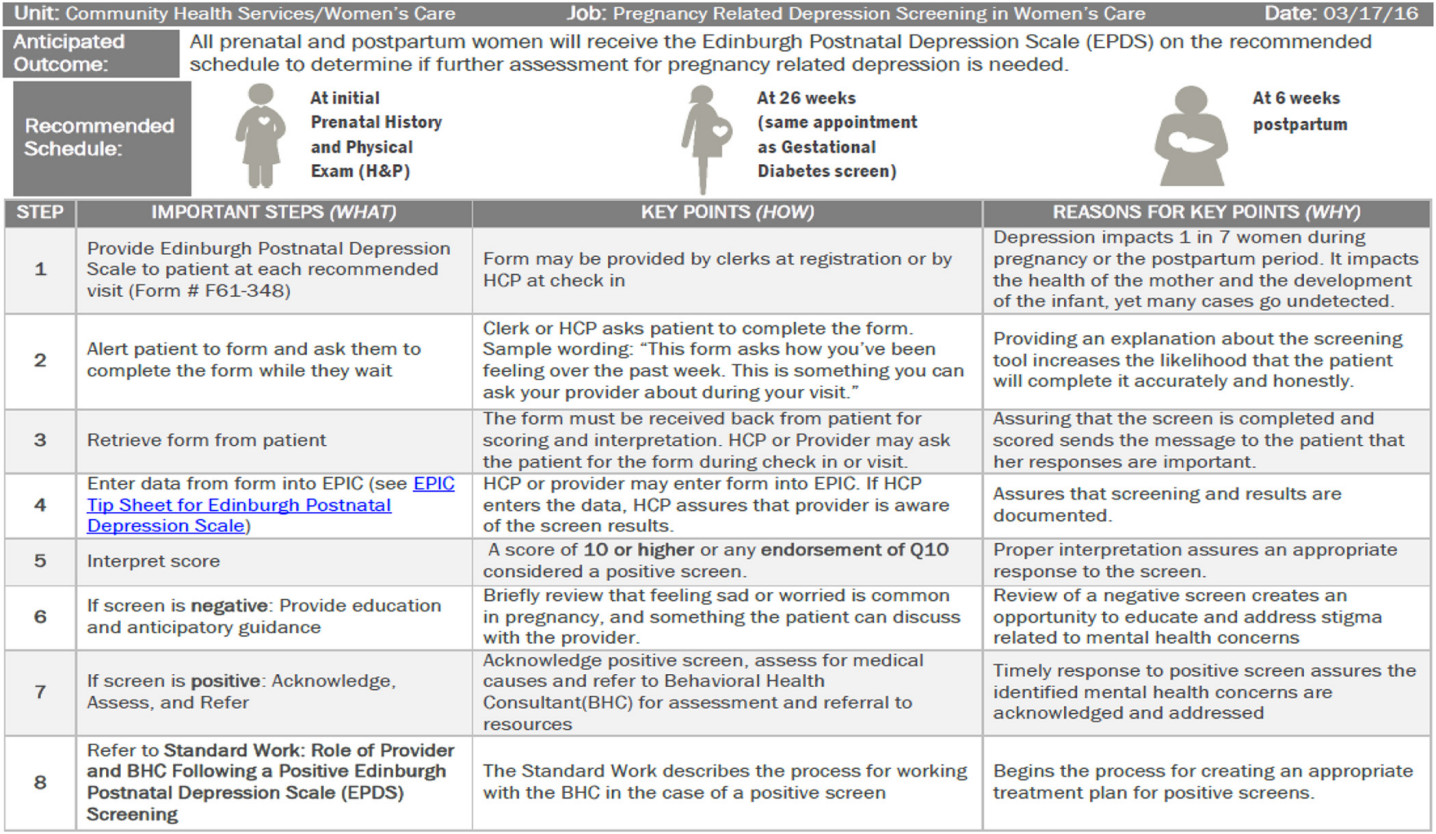
Standard work for universal screening for perinatal mood and anxiety disorders (PMADs) in perinatal clinics

In the fall of 2014, Denver Public Health department convened a multidisciplinary advisory board to guide the development and implementation of the screening program. The board was led and directed by a Lean facilitator. Lean is a systematic approach of continuous improvement utilized at Denver Health that uses a combination of principles and tools. Lean thinking is focused on: identifying and eliminating barriers, improving customer experience, and engaging the front line in improvement of work (Gabow, 2010). The advisory board developed a process map that described the current state of screening for PMADs. Through the mapping process, barriers were identified and processes developed to improve the consistency of screening for all women and the response to a positive screen. An example of the standard work that has been created for the universal screening process is presented in Figure 1.

Screening for PMADs is done initially at the OB intake visit by the medical assistant or nurse using the Edinburgh Postnatal Depression Scale (EPDS). The EPDS is a validated instrument used to assess depressive and anxiety symptomatology and suicidality in perinatal populations (EPDS: Cox *et al*. 1987). Despite its name, Edinburgh *Postnatal* Depression Scale, the instrument has been validated for use in pregnant populations and has been translated into over 50 languages (EPDS: Cox *et al*. 1987; Sit *et al*. 2009). Scores range from 0 to 30 with a score of greater than or equal to 10 and/or an endorsement of thoughts of self-harm ‘sometimes’ or ‘yes, quite often’ considered positive. A positive score prompts immediate referral to an imbedded, IBH provider at the same visit for evaluation and assessment of symptoms, needs, and treatment planning.

An EPDS is administered twice during pregnancy: at the time of the initial OB intake visit and at the beginning of the 3rd trimester. Generally, patients are screened at the 24–28-week point, at the time of gestational diabetes testing because it provides a memory prompt to perform the screen. This also allows the screening to become a routine part of their pregnancy and medical care, hopefully decreasing the stigma around mental health issues that arise in pregnancy and postpartum. Some patients are screened again at a two week postpartum mood check visit if they have (1) screened positive in the past, (2) have immediate postpartum symptoms, or (3) there are some other high-risk factors (drug abuse, social stressors). All women are screened at the six-week postpartum visit. Implementation also included involving pediatricians and family practice providers to screen mothers using the EPDS at the two, four, and six-month well child visits. This was implemented mainly due to our patient populations’ propensity to attend visits with their children, less so for themselves. Once a positive screen is determined, or if other cause for behavioral health concern is raised (eg, the patient’s affect is distressed or she reports distress) during a prenatal or well child care visit, same-day collaboration with a behavioral health provider occurs and may take multiple forms. This provider visit may include: ‘curb-siding,’ restructuring the visit to be an integrated or co-visit, or a provider-to-provider in-person patient hand-off. A behavioral health provider visit results in a detailed biopsychosocial assessment and, ultimately, establishment of follow-up behavioral health visits in tandem with future prenatal/postnatal care appointments. This type of collaboration allows for multi-functional assessments of a patient’s psychosocial experience, cognitive functioning, and behaviors that affect mood over the perinatal period.

Psychological intervention is provided within a brief, focused, short-term model of care. Women often prefer non-pharmacological interventions for treatment of perinatal mood disorders and those given a preference are more likely to engage in treatment (Dimidjian and Goodman, 2014). Treatment may be provided to individuals, couples, or families and the goal is to provide culturally sensitive, evidence-based psychotherapeutic care for our diverse population. Interventions are drawn from empirically validated treatments for prevention and treatment of perinatal mood disorders including but not limited to: cognitive behavioral therapy, interpersonal psychotherapy, mindfulness based cognitive therapy, acceptance and commitment therapy, solution focused therapy, and behavioral activation therapy (Dimidjian and Goodman, 2014; Stuart and Koleva, 2014). Goals of care include, but are not limited to, increasing social support, improving self-care, improved communication and problem solving, increasing pleasurable/value driven activities and increasing mindfulness (Stuart and Koleva, 2014).

Behavioral health providers also assist with referrals and case management as needed linking women with internal or external behavioral health or other supportive community resources. They are available to assist in the transition of the mother/baby dyad into primary care. Often the behavioral health providers in the women’s care perinatal clinic will coordinate directly with another behavioral health provider in the pediatric or family medicine clinic to ensure wrap-around coordination of services for the patient. Behavioral health consultants also have training in psychotropic medication and can provide psychoeducation and information about common side effects, how to take medications, and assist in medication compliance. The clinic psychiatrist can provide back up and consultation directly should the case require a more complex medication regimen.

**Fig. 2:**
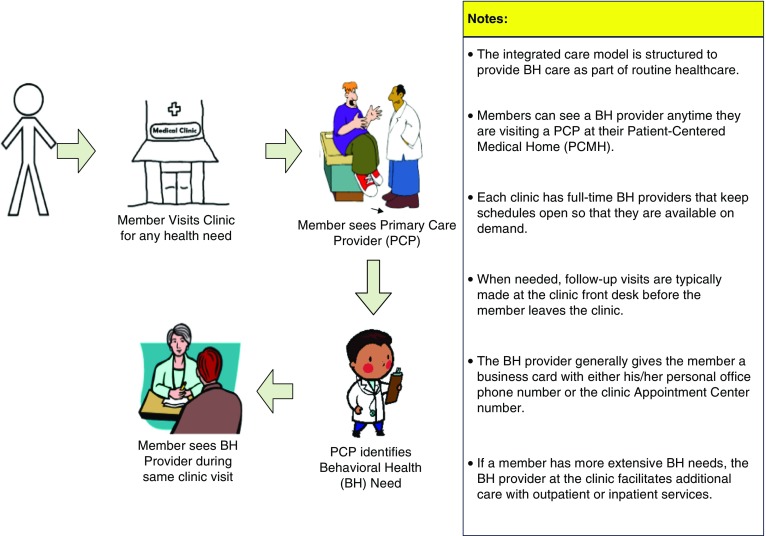
Integrated Behavioral Health (IBH) description

**Fig. 3:**
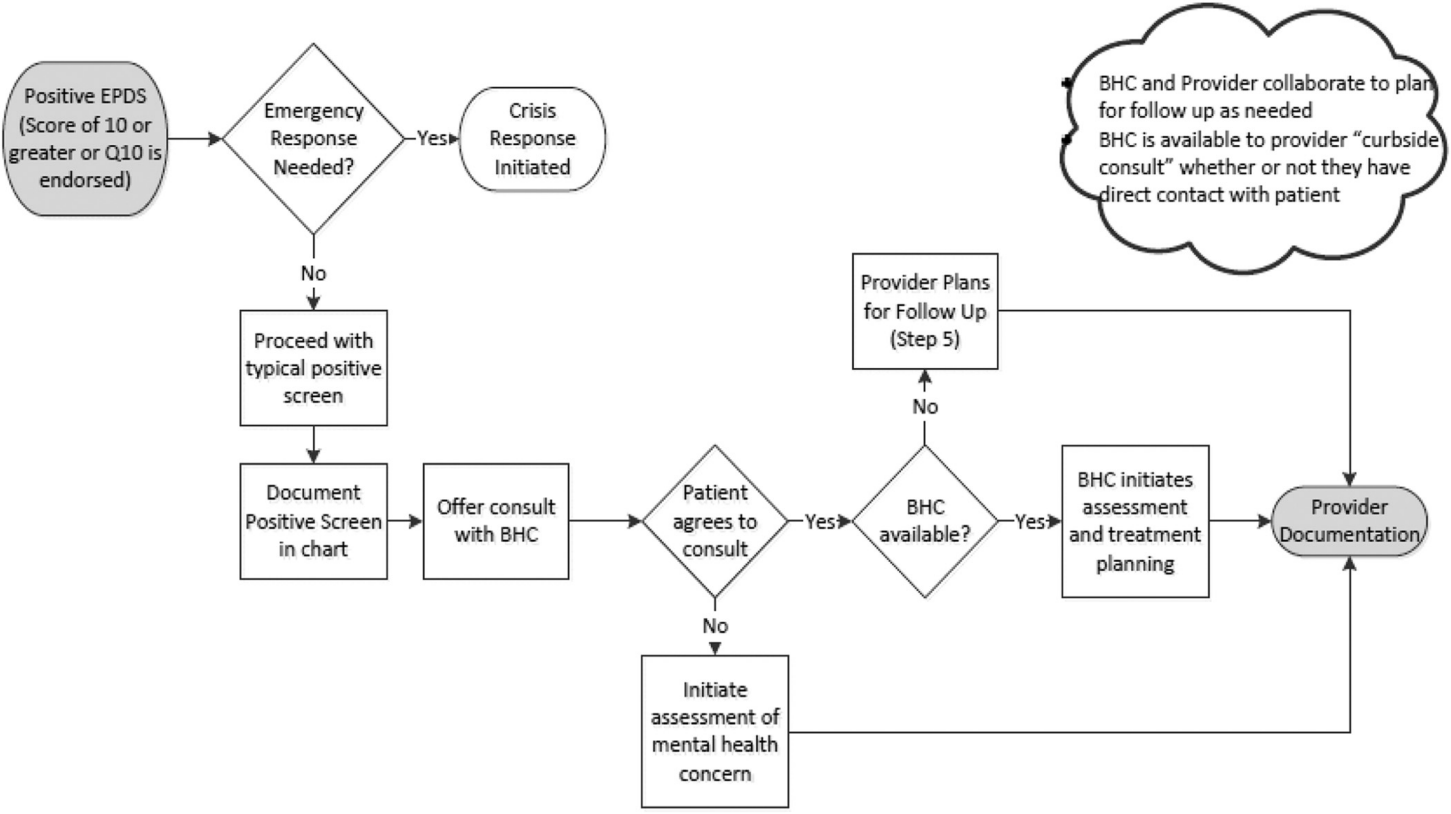
Process for responding to a ‘positive’ Edinburgh Postnatal Depression Scale screen. (Score of ⩾10)

If higher-level psychiatric care is necessary, a ‘Behavioral Health OB clinic,’ a schedule within the larger clinic where OB patients are seen by a nurse midwife and also by a psychiatrist and/or a clinical psychologist to manage more complex psychiatric issues in an integrated model. Additionally, a Perinatal Substance Use Disorder clinic was implemented in the fall of 2015 to specifically address and manage women and their infants with substance use disorders. Aided by the existence of these resources, the advisory board developed a multidisciplinary team response to a positive screen and this process is presented in Figure 2. As indicated in Figure 2, if the patient declines further consultation with a behavioral health provider for a positive screen or other concern, this is documented in the chart and the offer to meet with a behavioral health provider at subsequent visits is extended (Figure 3).

**Fig. 4:**
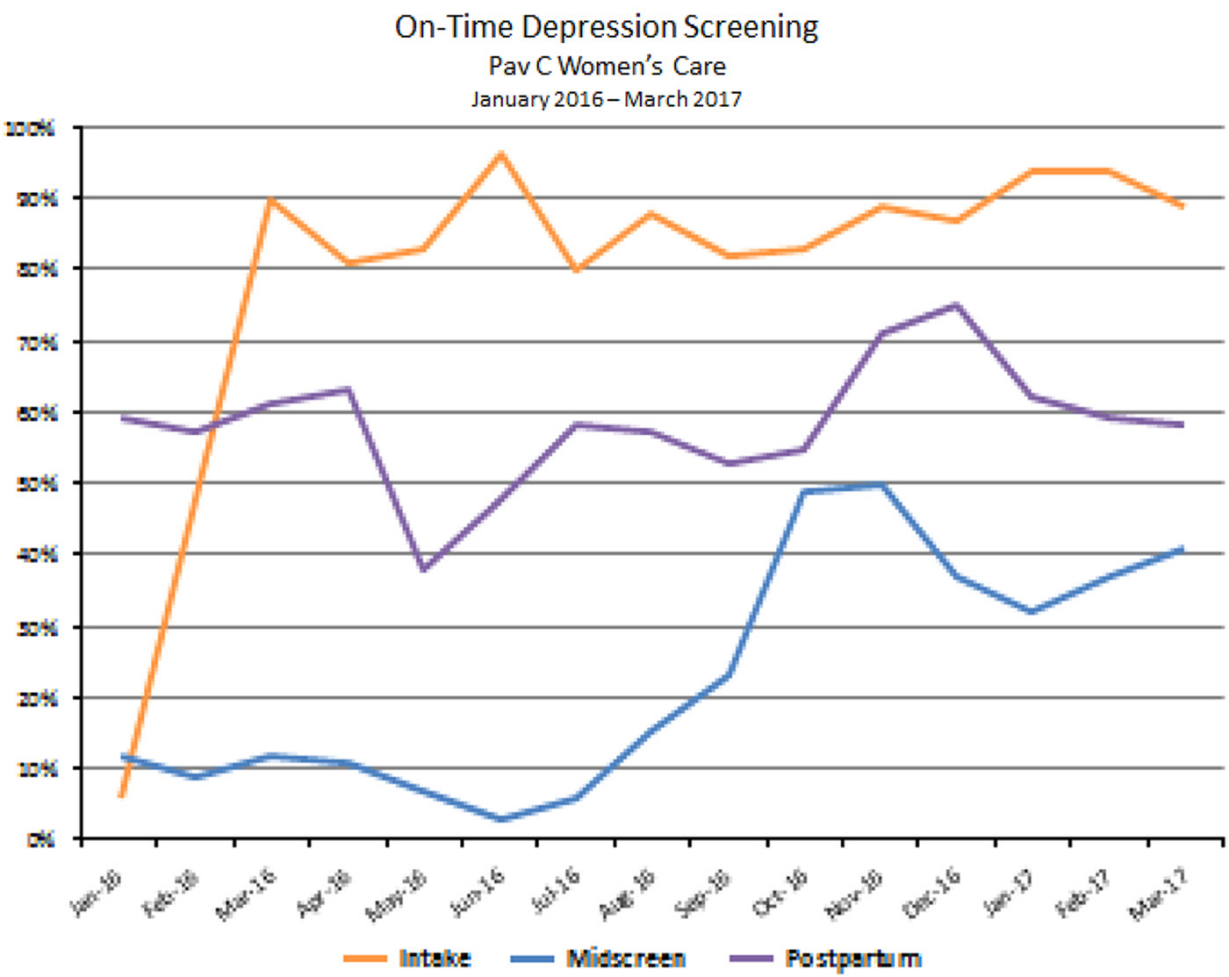
On time Edinburgh Postnatal Depression Scale (EPDS) screening in pregnancy in OB/Gyn clinic from January 2016 through March 2017

**Fig. 5:**
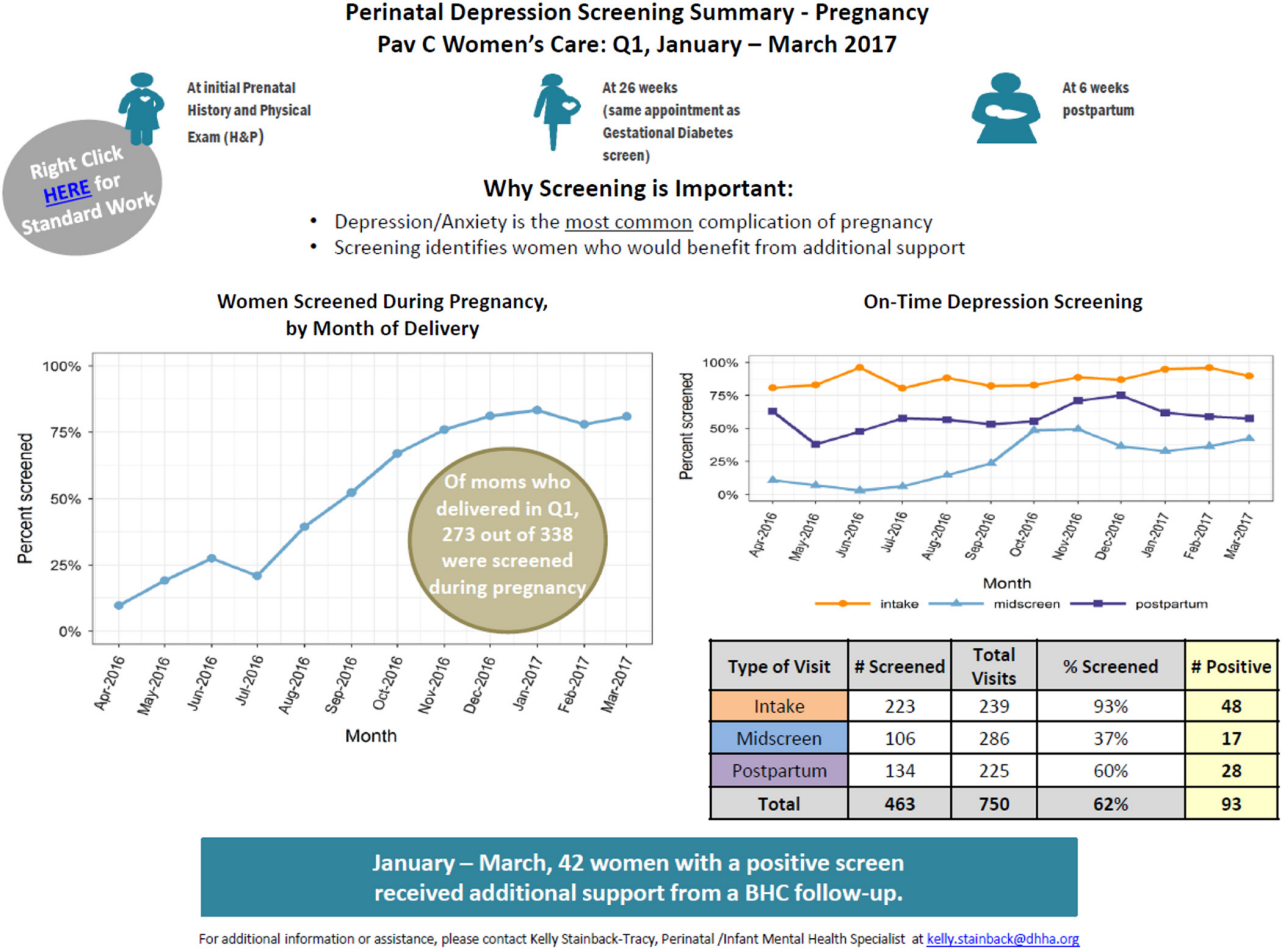
Trends in Edinburgh Postnatal Depression Scale (EPDS) screens at main OB/Gyn clinic from January 2016 through March 2017

**Fig. 6:**
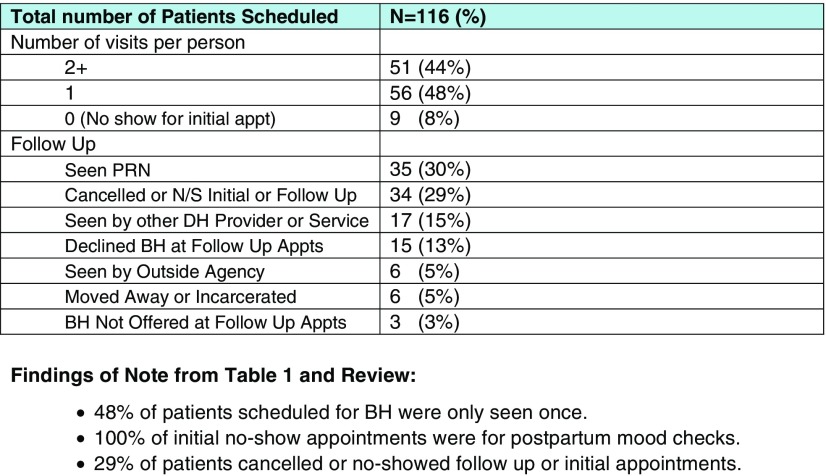
Behavioral health pregnancy related visits from July through October 2016

Since the onset of the program in August of 2014, the integrated perinatal mental health program has been implemented and expanded into seven community health and three Women’s Care led perinatal care clinics. All clinics are staffed with full-time behavioral health provider coverage for perinatal patients. As of June 2016, PMAD screening rates vary by clinic, ranging from 89% to 100% at the first prenatal care visit and 61–100% at the postpartum visit. These rates continue to improve from the initial tracking data in January 2015. Figure 4 represents the increase, over one year, of screening at the OB intake visit and at the postpartum visit at three clinics in the system. As more clinics within the system reach >90% of women screened, we will focus additional efforts on those clinics that are screening <90% of women and work to identify barriers and challenges at those clinic locations (Figures 5 and 6).

From August 2014 until December 2017, ~5350 behavioral health visits were billed that were co-occurring with a medical visit. Not all clinics have a coding process that elucidates a perinatal visit in tandem with an IBH visit, thus, this number is likely an underestimate. As seen in Figure 6, over a four-month period in one clinic, 116 different women were scheduled for behavioral health appointments; 48% of patients were seen one time, 44% were seen two or more times and 8% no-showed their initial appointment. Anecdotal feedback solicited from prenatal care providers has been overwhelmingly positive. In addition, enthusiasm for screening and implementation of screening has expanded rapidly now that the prenatal care team has a behavioral health provider present in all clinics for a same-day encounter and maternal mental health champions have been identified at all clinics. Identification of champions at each clinical site was an initial barrier to implementation of screening and to recruitment of behavioral health providers to cover all clinical sites. Another remaining challenge of this program is securing referral and transfer of a woman to community mental health resources when she is beyond the postpartum period. Figure 6 also demonstrated that women are still having difficulty utilizing behavioral health care with 29% of women canceling or no-showing their initial or follow-up visits.

This program, that integrates behavioral health into perinatal settings, meets the recommendations from a number of professional societies invested in improving maternal morbidity and mortality, improving perinatal mental health, and in expanding this type of integrated care (Agency for Healthcare Research and Quality, 2016). When perinatal mental health problems are addressed and treated, we see improvements in both pregnancy outcomes (Hoffman *et al*. 2016) and in multigenerational health and wellness (Stein *et al*. 2014). Systemic and social stigma around maternal mental health and PMAD’s is reduced by the introduction not only of screening but of treatment for these common concerns where women are engaged with trusted providers and in a trusted medical system. By introducing IBH into perinatal care, we have improved screening, assessment and management of complex mental and physical health issues during pregnancy and the postpartum period. Before implementation of this program, standard prenatal care primarily addressed the physical health of the mother, fetus, and infant. Because we were not screening for PMADs and not treating them, we were missing an important opportunity to address the most common complication of pregnancy. The implementation of this program has allowed us to address this concerning gap in the services we provide as we continually strive to improve the standard of care for perinatal care delivery.

We are hopeful that this program will serve the needs of women and families during this pivotal and transitional time in their lives and allow for behavioral change and openness to seek therapeutic intervention. In addition, as suicide and self-harm are the largest contributors to maternal mortality in the state of Colorado (Metz *et al*. 2016) we are hopeful that improving screening, assessment, and treatment during this vulnerable period will ultimately lead to a system that prevents long-term maternal and child morbidity as well as mortality.

We plan to provide ongoing evaluation of this program in order to further refine it and establish it as a recognized ‘best practice.’ In order to do this, we are tracking additional outcomes that include: the number of psychotherapy visits completed, initiation of pharmacotherapy and dose, diagnoses of postpartum depression or other psychiatric illness, gestational age at delivery, birth weight, and other delivery demographics. Long-term outcome measures include system-wide utilization of behavioral and physical health services by mothers and their children.

While we have implemented efforts to transition postpartum women with high-risk pregnancy conditions, including mental health issues, to primary care providers via patient navigation and social work, we are unable to serve every woman in need of ongoing care. We are therefore working on additional funds, both internally and externally, to secure long-term physical and behavioral health care for our patients. Secondarily Denver Health serves a large population of uninsured and/or undocumented women who may have financial challenges and therefore copays to see a behavioral health provider would be an undue burden. We are working with both grant providers and our Denver Health foundation to wave copays for pregnant and postpartum women to be able to have open access to the psychological care they may need. Another challenge is the known influence of childhood trauma, interpersonal violence, and substance dependence on the development of PMADs but the unknown prevalence of these issues in our population (Yonkers *et al*. 2014; Smith *et al*. 2006). We are working to determine the prevalence of these experiences and comorbidities and to develop a better understanding of how these influence both patterns of prenatal care utilization as well as the risk for prenatal and postnatal physical and mental health conditions.

Strengths of this program are primarily found in the interdisciplinary collaboration that moved this large effort forward and in the continued multidisciplinary standard work of running this program throughout our perinatal clinics. As well, the quantitative data shows that there is a decrease in reported depressive symptoms in moms who receive behavioral health support. As such, we see a decrease in ongoing toxic stress in the home which has the trickledown effect of improving the health and wellness of the family as whole. Given our ability to implement this program in a Federally Qualified Health Center, the use of this approach in clinics serving similar populations has the potential to improve maternal-child multigenerational health more broadly.
